# Polybenzoxazine-Based Nitrogen-Containing Porous Carbon and Their Composites with NiCo Bimetallic Oxides for Supercapacitor Applications

**DOI:** 10.3390/polym16030430

**Published:** 2024-02-03

**Authors:** Thirukumaran Periyasamy, Shakila Parveen Asrafali, Seong-Cheol Kim, Deivasigamani Ranjith Kumar, Jaewoong Lee

**Affiliations:** 1 Department of Fiber System Engineering, Yeungnam University, Gyeongsan 38541, Republic of Korea; 2 School of Chemical Engineering, Yeungnam University, Gyeongsan 38541, Republic of Korea; 3 Centre for Organic and Nanohybrid Electronics, Silesian University of Technology, Konarskiego 22B, 44-100 Gliwice, Poland

**Keywords:** hetero atom, porous carbon, bimetallic oxide, calcination temperature, electrode materials, SC application

## Abstract

Supercapacitors (SCs) are considered as emerging energy storage devices that bridge the gap between electrolytic capacitors and rechargeable batteries. However, due to their low energy density, their real-time usage is restricted. Hence, to enhance the energy density of SCs, we prepared hetero-atom-doped carbon along with bimetallic oxides at different calcination temperatures, viz., HC/NiCo@600, HC/NiCo@700, HC/NiCo@800 and HC/NiCo@900. The material produced at 800 °C (HC/NiCo@800) exhibits a hierarchical 3D flower-like morphology. The electrochemical measurement of the prepared materials was performed in a three-electrode system showing an enhanced specific capacitance for HC/NiCo@600 (Cs = 1515 F g^−1^) in 1 M KOH, at a current density of 1 A g^−1^, among others. An asymmetric SC device was also fabricated using HC/NiCo@800 as anode and HC as cathode (HC/NiCo@600//HC). The fabricated device had the ability to operate at a high voltage window (~1.6 V), exhibiting a specific capacitance of 142 F g^−1^ at a current density of 1 A g^−1^; power density of 743.11 W kg^−1^ and energy density of 49.93 Wh kg^−1^. Altogether, a simple strategy of hetero-atom doping and bimetallic inclusion into the carbon framework enhances the energy density of SCs.

## 1. Introduction

In the last few decades, the demand for alternative high-performance energy storage devices has increased enormously due to the global energy crisis and environmental pollution. Due to this increasing demand, extensive research has been focused on portable and wearable electronic energy storage technologies, energy harvesting devices and hybrid electric vehicles [1,2,3,4]. In the field of portable electronics, lithium-ion batteries (LIBs) have been widely used as energy storage materials for several years due to their high energy density and long cyclic stability. However, they have safety issues as liquid electrolytes are employed in LIBs. Therefore, developing a stable and reliable energy storage device without sacrificing the electrochemical performance is of primary concern [5,6,7,8]. In recent years, supercapacitors (SCs) have also been used as important energy storage devices due to their unique characteristics, including high power density, excellent energy efficiency and extremely long cyclic stability, high charge–discharge rate and environment friendliness [9,10,11,12]. Based on their energy storage and energy delivering capacities, SCs find themselves superior to capacitors and batteries as they can store higher energy densities than capacitors and can manage higher power densities than batteries. Thus, SC devices are an optimal option where short charge/discharge time, moderate charge storage and high reliability are required [13,14,15]. Trending today, the world is moving towards the usage of wearable mini-electronic devices and many research efforts have been focused on thin, small and flexible SCs with high energy and power densities. In spite of this great progress, flexible SCs still suffer from poor energy output and long cyclic stability that limits their application. And therefore, proper design of the structure of electrode materials in SCs and the stability of electrolytes are the primary criteria in their development [16,17,18,19,20,21].

There are two ways to increase the energy density of SCs. Firstly, increased energy density can be obtained by increasing the specific capacitance of the electrode materials, which could be attained by incorporating both double-layer capacitance and pseudo-capacitance. Secondly, the energy density of SCs can be improved by increasing the voltage range of the cell, as is evidenced by the following equation given below,

(1) $$E={1/2CV}^{2}$$

where ‘E’ is the energy density; ‘C’ is the specific capacitance and ‘V’ is the voltage range. Moreover, in considering the durability of the solid-state SCs, the thickness of the electrode materials plays an active role as the usage of active materials generally decays to a larger extent with thick electrodes [22,23].

Presently, most of the commercial electrode materials used in SCs are purely carbon-based materials and hence, the charge storage mechanism is purely electrostatic, produced by electrical double-layer capacitance (EDLC). And so, when the electrode materials are constructed with both EDLC and pseudo-capacitance, a further enhancement in energy and power density can be obtained, as pseudo-capacitive materials enhance the specific capacitance of the electrode materials electrochemically (i.e., by interfacial reversible Faradaic reactions). A variety of organic, inorganic and polymeric substances have been used as electrode materials for SCs, which includes activated carbons, carbon nanotubes (CNTs), transition metal oxides/sulfides/hydroxides and conducting polymers. The choice of these materials is governed by certain factors including specific surface area, pore size, 3D hierarchical structure, additional redox reactions and electrical conductivity of the electrode active materials [11,12,23,24].

Transition metal oxides/hydroxides, such as Ni, Co and Mn oxides/hydroxides are considered promising electrode materials for SCs on account of their reversible oxidation reduction states, cost effectiveness and environment friendliness. Moreover, bimetallic oxides are more preferred than their monometallic parts [25], as the former can produce multiple oxidation states, producing more redox reactions and resulting in high pseudo-capacitance [10,18,21]. Chen et al. [19] synthesized a composite material containing a conductive polymer and metal-organic framework through the hydrothermal method. The synthesized material, PPy@NiCo-CAT (polypyrrole with NiCo-catecholate), when used as an electrode material in a hybrid supercapacitor, exhibits an energy density of 22 Wh kg^−1^ and a power density of 400 W kg^−1^. Xiao et al. [25] used shell-coating and a controlled etching process to construct a non-spherical hollow bimetal phosphate nanocage, ZIF-67-LDH-CNP-110. They also showed that the hybrid capacitor ZIF-67-LDH-CNP-110//AC showed a remarkable energy density of 33.29 Wh kg^−1^ and a power density of 150 W kg^−1^. Khalafallah et al. [26] synthesized WPP (waste potato peel)-derived hierarchical porous carbon with heteroatom doping derived from hypophosphite and thioacetamide (S, P/PAC). When used as electrode materials, (S, P/PAC) showed a high specific capacitance of 323 F g^−1^ at 1 A g^−1^, a high energy density of 45.5 Wh kg^−1^ and a power density of 800 W kg^−1^. With respect to carbon materials in SC, several modifications have been performed to produce hetero-atom-doped carbon for enhanced performance. In this regard, polybenzoxazine (Pbz), when used as the carbon precursor, can easily induce nitrogen and oxygen doping into the carbon framework. Polybenzoxazines are an advanced class of phenol-formaldehyde resin possessing several unique characteristics, including low surface free energy, low moisture absorption, molecular design flexibility and excellent mechanical and dimensional stability. The carbon prepared from this Pbz is found to be rich in nitrogen and oxygen species that can preferably enhance the electrochemical performance of the SCs [27,28,29].

In this work, we propose a strategy to produce electrode materials with carbon materials (from Pbz) and bimetallic transition metal oxides (oxides of Ni and Co), where their synergistic effect can produce both EDLC and pseudo-capacitance, which could enhance the overall performance of SCs. The novelty of the work lies in choosing polybenzoxazine as a source of carbon so that heteroatoms like nitrogen and oxygen will be incorporated in the carbon framework. And the inclusion of bimetal oxides into this carbon framework paves way for increased capacitance due to the double-layer and pseudo-capacitance. Apart from the inclusion of these materials, the processing temperature, i.e., the activation temperature, also plays an importance role in the performance of the SC. Hence, four different activation temperatures (i.e., 600, 700, 800 and 900 °C) have been chosen to produce the carbon materials. The effects of these four different activation temperatures in enhancing the specific capacitance and electrochemical performance of the electrode material were analyzed in the three-electrode system. For practical applications, a two-electrode system was assembled for the best electrode material and its electrochemical performance, including energy and power density, was calculated and reported.

## 2. Materials

Eugenol (99%) and paraformaldehyde (95%) were purchased from Sigma-Aldrich (St. Louis, MO, USA). Ethylene diamine (99%), potassium hydroxide (KOH, 85–87%), sodium hydroxide (NaOH, 97%) and dimethyl sulfoxide (DMSO, 99%) were purchased from Duksan Chemicals Co., Ltd. Ansan-si, Republic of Korea. Nickel nitrate hexahydrate (99%) cobalt nitrate tetrahydrate (97%), polyvinylidene fluoride (PVDF, 97%) and N, N-dimethylformamide (DMF, 99.5%) were purchased from Duksan Chemicals Co., Ltd., Ansan-si, Republic of Korea. All chemicals were used without further purification.

## 3. Methods

### 3.1. Synthesis of Hetero-Atom-Doped Carbon Materials

The synthesis of hetero-atom-doped carbon materials was performed through four different processes, viz., benzoxazine monomer synthesis, self-curing polymerization to form polybenzoxazine, carbonization and, finally, activation. Polybenzoxazine was chosen as a source to produce carbon materials as polybenzoxazine contains hetero atoms (such as nitrogen and oxygen) inside the cyclic ring structure, which, when carbonized, resulted in the formation of hetero-atoms doped inside the carbon framework. The benzoxazine monomer synthesis followed a Mannich condensation process, where eugenol (phenolic moiety), ethylene diamine (amine moiety) and paraformaldehyde underwent a condensation reaction to form eugenol-based benzoxazine (Eu-Bzo). Then, the formed monomer was subjected to a self-curing process (up to 250 °C) where the polymer (polybenzoxazine) was produced. The polymer was subjected to carbonization (800 °C, N_2_ atm.) and activation in the presence of KOH (twice the amount of the carbon material) at 800 °C in N_2_ atm., with a heating rate of 2 °C/min and a holding time of 1 h in a tubular furnace to produce the hetero-atom-doped carbon materials, abbreviated as HC.

### 3.2. Synthesis of HC/NiCo Materials

**Scheme 1:**
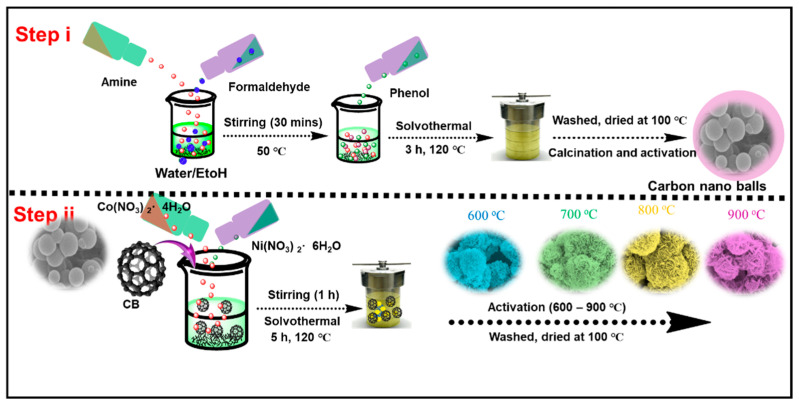
Schematic diagram of the preparation of HC/NiCo at different temperatures.

The prepared HC materials (10 mg), along with the precursors of Ni [Ni(NO_3_)_2_·6H_2_O] (12 mM) and Co [Co(NO_3_)_2_·4H_2_O] (6 mM) in a 2:1 ratio were dispersed in a mixture of solvents containing H_2_O, DMF and ethanol (equal volumetric ratios) for 1 h to attain complete dispersion of the materials. The resulting mixture was then subjected to a solvothermal reaction at 120 °C for 24 h, maintaining the volume of the solvents as one-third the volume of the autoclave, and then washed several times with DMF and ethanol, before finally being filtered and dried completely to form the HC/NiCo material. The prepared HC/NiCo material was then calcined at four different temperatures (i.e., 600, 700, 800 and 900 °C) in a tubular furnace under N_2_ atmosphere to produce HC/NiCo@600, HC/NiCo@700, HC/NiCo@800 and HC/NiCo@900, respectively (Scheme 1).

### 3.3. Fabrication of Working Electrode

The working electrodes for the electrochemical measurement were fabricated by following the method given below. Each of the prepared material(s) [HC/NiCo@600, HC/NiCo@700, HC/NiCo@800 and HC/NiCo@900] was mixed with PVDF (ratio of carbon material and PVDF is 95:5) and NMP (up to 5 mL) to form a homogeneous paste. The paste was then coated uniformly on both sides of the nickel foam and dried completely. These were used as working electrodes and several electrochemical measurements were taken with this.

## 4. Results and Discussion

The 3D hierarchical flower-like morphology of HC/NiCo@800 was obtained in two different steps. In the first step, a solvothermal reaction at 120 °C for 24 h resulted in the introduction of bimetallic components into the carbon framework. In the second step, HC/NiCo was converted into an integrative 3D hierarchical flower by calcination at 800 °C. The calcination temperature varied between 600 and 900 °C, and we found that 800 °C was suitable to form the proper 3D structure. Hence, the calcination temperature plays an important role in the formation of the 3D hierarchical structure. The formed HC/NiCo at different calcination temperatures (say 600, 700, 800 and 900 °C) was further characterized for preliminary analysis of structure and composition. Scheme 1 represents the detailed synthesis procedure of synthesized-PBz and HC.

The structural characterization of the synthesized materials, HC/NiCo@600; HC/NiCo@700; HC/NiCo@800 and HC/NiCo@900 at different calcination temperatures, including XRD, Raman, BET and XPS, are analyzed and discussed in the supporting information.

### 4.1. Morphological Studies

#### 4.1.1. SEM Analysis

**Figure 1:**
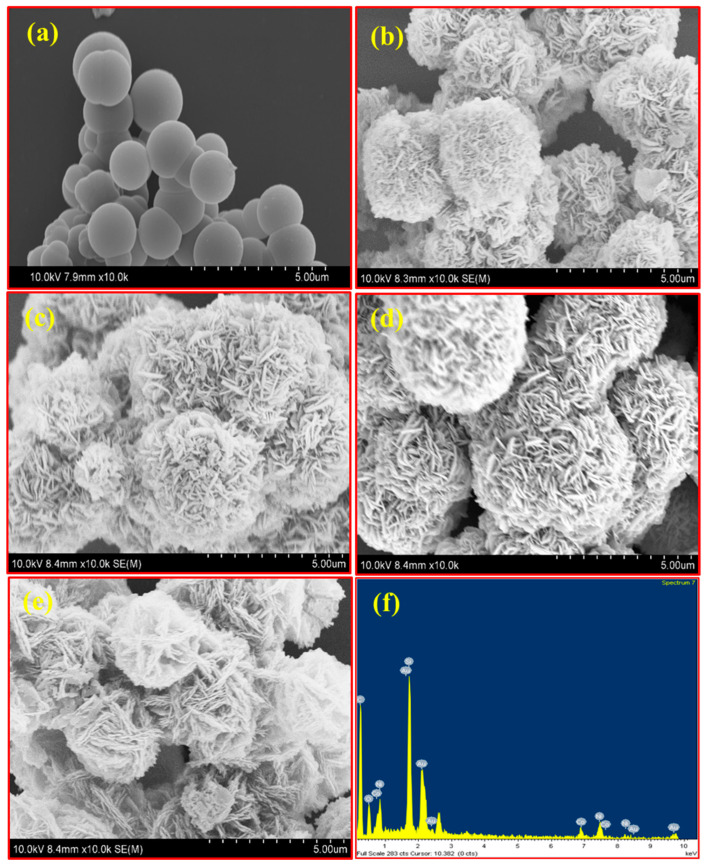
SEM images of (**a**) HC; (**b**–**e**) HC/NiCo at different calcination temperatures (600–900 °C); and (**f**) EDX spectrum of HC/NiCo@800.

The morphology of HC/NiCo formed at different calcination temperatures was analyzed using FESEM. Figure 1a–f portrays the SEM images of pristine carbon, HC/NiCo, at different calcination temperatures and the EDX spectrum of HC/NiCo@800. As can be seen from the image (Figure 1a), the pristine carbon has a spherical shape with a smooth texture. It can be visualized that several spherical balls with varying sizes are joined together. Soon after the incorporation of bimetallic oxides and the calcination process, the morphology of the materials is completely changed from a spherical shape to a flower shape (Figure 1b–e). It can be seen that the flower morphology was produced in this way: the carbon material forms the basic spherical structure, upon which the bimetallic oxides, as 2D petals, grow together, forming a 3D hierarchical flower-like structure. The importance of calcination temperature is clearly evident from the SEM images. A calcination temperature of 600 and 700 °C (Figure 1b,c) is not enough to form a proper 3D structure as the flowers are not formed properly, resulting in broken petals here and there, whereas at 800 °C (Figure 1d), a complete 3D hierarchical flower is formed, where several petals are in intact, forming a complete structure. At a still higher calcination temperature (say 900 °C), the petals are clubbed together, leaving a lot void space, showing withered flower morphology (Figure 1e). Moreover, some flower structure have been already destroyed, with several non-uniformities in their structure. This structural evidence confirms that a calcination temperature of 800 °C is appropriate to form a flower-like morphology. This kind of entangled porous network structure, consisting of several spheres onto which there is vertical alignment of 2D petals, facilitates charge transport and ion diffusion without any interruption. The EDX spectrum of HC/NiCo@800 in Figure 1f also confirms the presence of all the elements (i.e., C, N, O, Co and Ni) in their respective ratios [10,18,30,31].

#### 4.1.2. TEM Analysis

**Figure 2:**
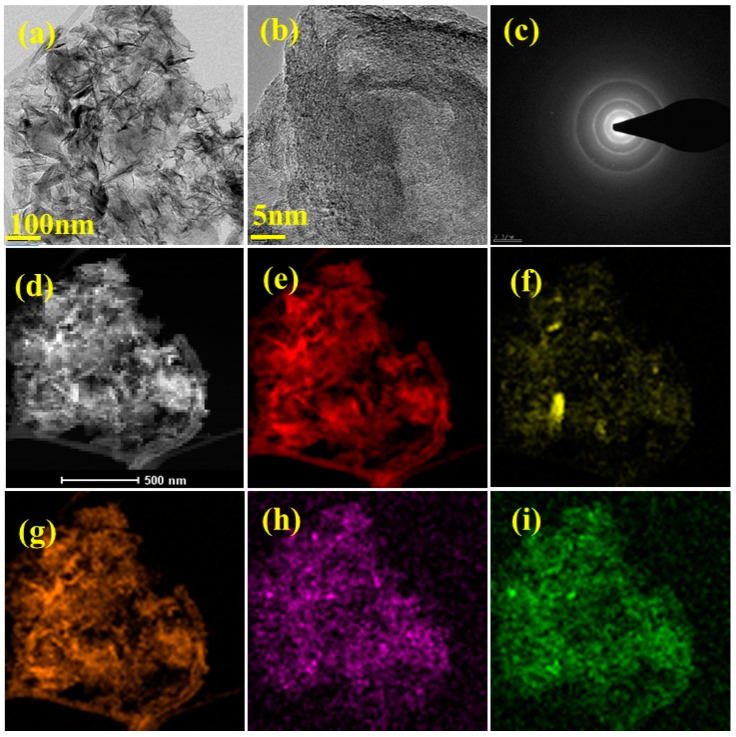
(**a**,**b**) HR-TEM images of HC/NiCo@800 along with (**c**) SAED pattern and (**d**–**i**) elemental mapping.

A more detailed surface integration of HC/NiCo@800 was further verified using HRTEM. Figure 2a–i depict the HRTEM images along with the SAED pattern and EDS mapping of HC/NiCo@800. The hierarchical structure with two different morphologies can be clearly seen from the TEM image (Figure 2a). The hierarchical structure consists of an ultra-thin, porous structure that forms the base, upon which sharp spikes are embedded throughout uniformly. The porous structure is derived from the spherical shape (of carbon) and the spike-like structure is derived from the petal shape (of bimetallic oxides), which is in good agreement with the SEM results. The highly magnified image in Figure 2b shows that the structure is densely packed without any aggregation. This densely packed structure, containing conductive carbon material onto which the bimetallic oxides are tightly anchored, will provide highly active interfaces and redox centers for enhanced supercapacitor performance. Moreover, the different phases of bimetallic oxides are evident from the SAED pattern (Figure 2c). The EDS mapping confirms the presence of all elements, i.e., C, N, O, Co and Ni, with uniform distribution. This further implies that Co and Ni can be uniformly embedded in the carbon framework (Figure 2d–i). All the preliminary characterizations confirm that HC/NiCo@800 has been successfully prepared with the desired morphology suitable for electrode materials in SC applications [10,12,18,22].

### 4.2. Electrochemical Results

#### 4.2.1. Three-Electrode System

**Figure 3:**
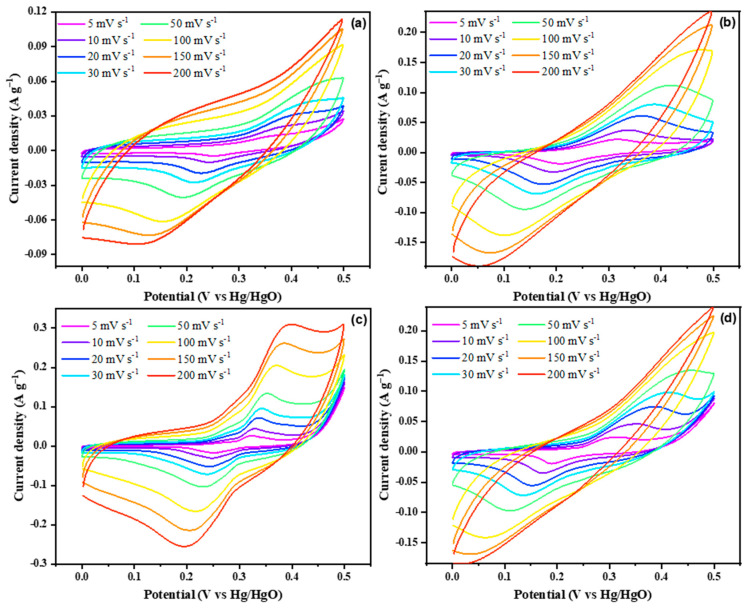
CV graphs of (**a**) HC/NiCo@600; (**b**) HC/NiCo@700; (**c**) HC/NiCo@800 and (**d**) HC/NiCo@900.

**Figure 4:**
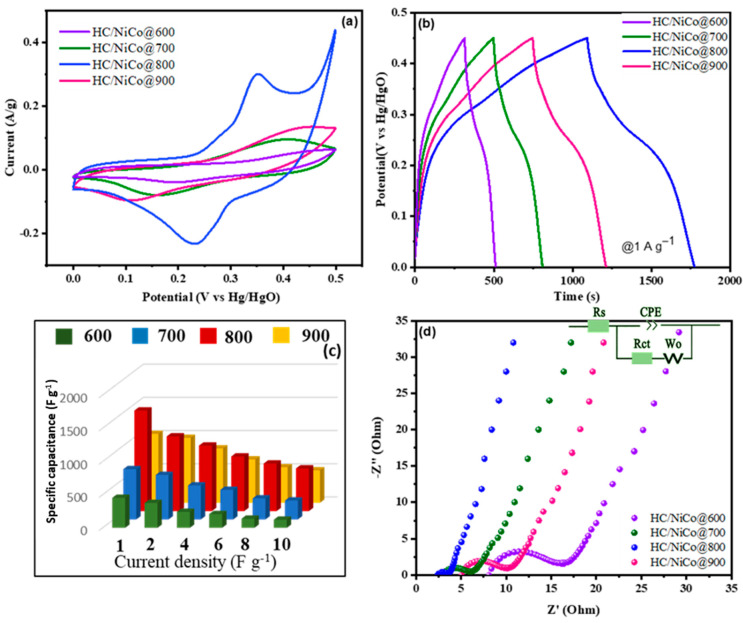
(**a**) CV graphs of HC/NiCo; (**b**) GCD graphs of HC/NiCo; (**c**) specific capacity of HC/NiCo; and (**d**) EIS spectra of HC/NiCo.

To investigate the charge-storage kinetics, the as-synthesized materials, i.e., HC/NiCo, at different calcination temperatures were used as electrode materials and their electrochemical behavior was investigated by cyclic voltammetry (CV), galvanostatic charge–discharge (GCD) and electrochemical impedance spectra (EIS) in a three-electrode system in a 1M KOH electrolyte, using Hg/HgO as a reference electrode and Pt as a counter electrode. A detailed fabrication of the working electrode is given in supplementary information. Figure 3a–d represent the CV curves of all the prepared electrode materials at different scan rates from 5 to 200 mV s^−1^. The CV curves of HC/NiCo@600, HC/NiCo@700 and HC/NiCo@900 (Figure 3a,b,d) show a quasi-rectangular shape at a high scan rate and a very small redox peak at lower scan rates, whereas for HC/NiCo@800 (Figure 3c), a redox peak was observed with an oxidation peak at 0.4 V and a reduction peak at 0.2 V at all scan rates. The significant redox peak even at a high scan rate of 200 mV s^−1^, indicates the pseudo-capacitance behavior of the electrode materials [32,33,34,35,36]. This clearly shows that a calcination temperature of 800 °C is suitable for the incorporation of bimetallic oxide into the carbon material, as evidenced by XRD and Raman analyses (Figure S1a,b). Moreover, the shape of the CV curve is well maintained with an increasing scan rate (Figure 3c), indicating reversible and fast ion and charge transfer capability collectively due to the porous structure (Figure S1c,d) and flower-like morphology (Figure 1). Figure 4a–d show the CV curves at a scan rate of 30 mV s^−1^, GCD at a current density of 1 A g^−1^, specific capacity at various current densities (1–10 A g^−1^) and impedance spectra of the prepared materials. As can be seen from the figure (Figure 4a), the area within the CV curve is much larger for the samples HC/NiCo@800 and HC/NiCo@900, indicating high capacitance in the form of EDLC (electrical double-layer capacitance) and pseudo-capacitance. This large CV area is very much reciprocated in their GCD curves. All the GCD curves possess non-equilateral triangles due to the presence of Faraday behavior, in addition to double-layer behavior [37,38,39,40,41,42] (Figure 4b). As expected, HC/NiCo@800 exhibits the longest discharge time at 1 A g^−1^, when compared with the other three materials. The specific capacitance (C_s_) at different current densities was calculated from the GCD curves, using equation [2].

(2) $$Cs=\frac{I\times \Delta t}{m\times \Delta V}$$

where C_s_ is the specific capacitance of the material; I is the current; m is the mass of the material; ∆t is the discharge time and ∆V is the voltage range.

The C_s_ at 1 A g^−1^ was calculated to be 453 F g^−1^ for HC/NiCo@600; 760 F g^−1^ for HC/NiCo@700; 1515 F g^−1^ for HC/NiCo@800 and 1035 F g^−1^ for HC/NiCo@900 (Figure 4c). Among the four electrode materials, HC/NiCo@800 has the highest C_s_ value that could be maintained up to 644 F g^−1^ even at a higher current density of 10 A g^−1^. This highest C_s_ value confirms the enhanced electrochemical performance of HC/NiCo@800.

The impedance of the electrode materials at different frequencies from 0.01 Hz to 100 kHz was measured and is presented in Figure 4d. All the EIS spectra show Nyquist plots showing a semi-circle in the medium- to high-frequency region and a straight line in the low-frequency region. The solution resistance (R_S_, starting point for the semi-circle) and the charge-transfer resistance (R_CT_, diameter of the semi-circle) are obtained from the Nyquist plots. The R_S_ and R_CT_ of the prepared electrode materials were found to be 8.09 and 7.31 Ω for HC/NiCo@600; 3.13 and 2.84 Ω for HC/NiCo@700; 2.43 and 1.15 Ω for HC/NiCo@800 and 5.05 and 4.91 Ω for HC/NiCo@900. The lowest value of R_S_ and R_CT_ for HC/NiCo@800 (2.43 and 1.15 Ω) indicates an efficient electrolyte diffusion and faster ion-transport rate that significantly improves the electronic conductivity. Moreover, the angle formed between the semi-circle and Warburg impedance is more inclined for HC/NiCo@800 with an angle of 75°, when compared with the other electrodes, indicating the faster diffusion rate of the cationic species [43,44].

**Figure 5:**
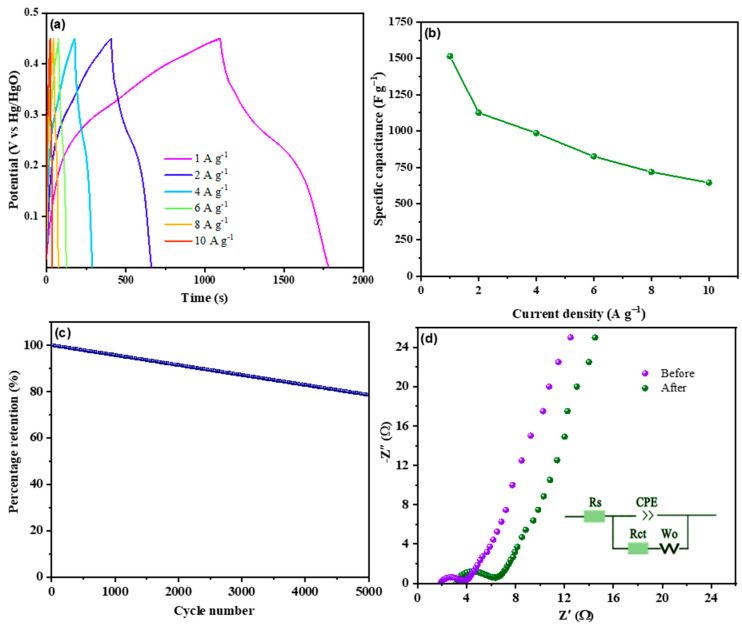
(**a**) GCD; (**b**) specific capacitance; (**c**) cyclic stability; and (**d**) EIS spectra of HC/NiCo@800.

As the HC/NiCo@800 electrode possess the highest C_s_, its stability is verified by the cyclic rate performance. Figure 5a,b depict the stability of the HC/NiCo@800 electrode at different current densities and Figure 5c displays the capacitance retention of the HC/NiCo@800 electrode at a current density of 1 A g^−1^ for over 5000 cycles. The capacitance retention of the electrode was found to be 78.5% even after 5000 cycles, implying remarkable cyclic stability. The morphology of the electrode slightly collapsed after stability measurements, as evidenced by the SEM images (Figure S3). The EIS was carried out for the 1st and 5000th cycle and is displayed in Figure 5d. A slight increase in R_S_ value from 1.89 to 3.27 Ω and R_CT_ value from 1.76 to 2.95 Ω was observed, indicating excellent capacitance retention [12,17].

#### 4.2.2. Performance of Asymmetric Supercapacitor Device

To further explore the practical application of HC/NiCo@800, we constructed an asymmetric supercapacitor (ASC) device, i.e., HC/NiCo@800//HC, in which HC/NiCo@800 was employed as a positive electrode and HC was employed as a negative electrode. Both the electrodes were coated separately onto a Ni foam and separated using a tissue paper. These electrodes were well wetted with 1 M KOH electrolyte solution. The electrochemical measurements, including CV, GCD, cyclic stability and EIS, for this device were measured and its energy and power density were calculated.

**Figure 6:**
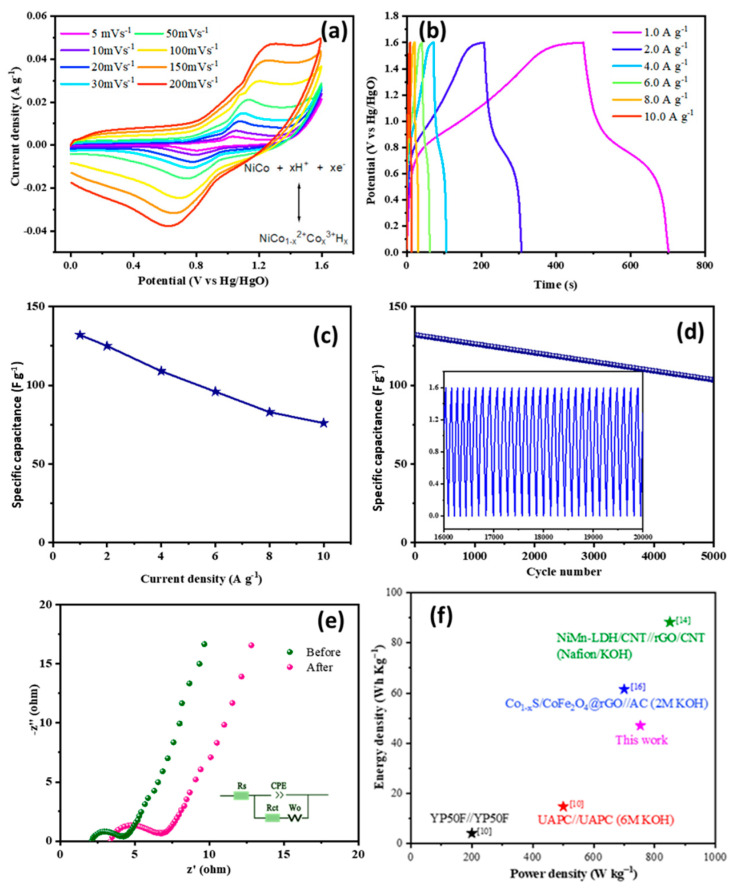
(**a**) CV; (**b**) GCD; (**c**) specific capacity; (**d**) cyclic stability; (**e**) EIS spectra; and (**f**) Ragone plot of HC/NiCo@800//HC.

Figure 6a shows the CV curves of the ASC device at different scan rates from 5 to 200 mV s^−1^. The operating voltage of ASC was fixed between 0 and 1.6 V, as the HC negative electrode showed potential range between −1.0 and 0 V and the HC/NiCo@900 positive electrode showed potential range between 0 and 0.6 V in a three electrode system (Figure S4). Noticeable redox peaks were observed for the ASC device due to the oxidation (at 1.25 V) and reduction (at 0.63 V) reactions. These redox peaks are attributed to the pseudo-capacitance produced from both the hetero-atom-doped carbon and bimetal oxides. Good shape retention of the CV curves was obtained even at high scan rates, suggesting excellent stability and fast charge/discharge performance of the electrode materials. Moreover, the area within the CV curve was also increased with increasing scan rates, and the voltage window was also increased up to 1.6 V, which could contribute to enhanced capacitance performance. Figure 6b depicts the GCD curves of the ASC device at different current densities from 1 to 10 A g^−1^. All the GCD curves are non-linear, suggesting both pseudo-capacitance and EDLC [45,46,47]. Obviously, the corresponding voltage plateaus of GCD are almost identical to the CV curves, indicating good agreement between the two.

The C_s_ calculated from the discharge time of GCD was found to be 132 F g^−1^ at a current density of 1 A g^−1^. A specific capacitance of 76 F g^−1^ was obtained even at a higher current density of 10 A g^−1^ (Figure 6c). The decreased C_s_ value at the high current density is due to the fact that there is difficulty in the adsorption and diffusion of ions into the small pores at higher currents. The durability of the device was examined through GCD measurements over 5000 cycles, at a current density of 1 A g^−1^. The obtained C_s_ values for over 5000 cycles are displayed in Figure 6d. The ASC device displayed good cyclic stability, maintaining a C_s_ of 103 F g^−1^ after 5000 cycles, showing a capacitance retention of 78.48%. The EIS was performed before and after the cyclic stability and is displayed in Figure 6e. The Nyquist plot shows a semicircle in the high-frequency region, in which both the solution and charge-transfer resistance have been increased (R_S_ from 2.10 to 3.37 Ω and R_CT_ from 1.96 to 3.37 Ω) after the cyclic stability measurements. The semicircle at the high-frequency region is due to the combination of solution resistance and charge-transfer resistance, whereas the linear region in the low frequency region is due to the Warburg diffusion process. The energy density and power density of the ASC device were calculated using Equations (3) and (4), and a Ragone plot was constructed (Figure 6f).

(3) $$E=\frac{{CV}^{2}}{2}$$

(4) $$P=\frac{E}{\Delta t}$$

where E and P are the energy and power densities; C is the specific capacitance; V is the voltage and ∆t is the discharge time. The ASC device exhibits an energy density and power density of 49.93 Wh kg^−1^ and 743.11 W kg^−1^ at a current density of 1 A g^−1^, and an energy density and power density of 27.02 Wh kg^−1^ and 427.89 W kg^−1^ at a current density of 10 A g^−1^. The comparison data with respect to energy and power densities are given in Figure 6f. Hence, the improved electrochemical performance is due to the combined effect of hetero atoms and bimetallic oxides in the interconnected carbon framework that improves the wettability of the electrode materials and provides pseudo-capacitance in addition to EDLC [12,21,48,49]. There is an increase in the voltage window and discharge time, which further increases the C_s_, resulting in increased energy density without sacrificing the power density, as well.

## 5. Conclusions

In summary, HC/NiCo@800 was successfully fabricated using a simple and versatile method, i.e., the solvothermal method and calcination. A calcination temperature of 800 °C is effective in creating a 3D hierarchical flower-like structure with a uniform porous structure. The BET analysis showed that the maximum pores are in the microporous and mesoporous range, which is suitable for electrochemical applications. The prepared HC/NiCo@800, when used as an electrode material in a three-electrode system showed enhanced capacitance of 1515 F g^−1^, which is solely attributed to the pseudo-capacitance in addition to EDLC. In addition to it, the impedance measurements display a lower solution resistance (1.89 Ω) and charge-transfer resistance (1.76 Ω), indicating the high conductivity of the prepared materials. The practical application of this electrode material is demonstrated by fabricating a two-electrode device with HC/NiCo@800 as the anode and HC as the cathode. The asymmetric device exhibits a specific capacitance of 132 F g^−1^ at a current density of 1 A g^−1^ and holds a capacitance retention of 78.48% over 5000 cycles. The interconnected 3D hierarchical porous structure formed from nitrogen and oxygen containing porous carbon together with bimetallic oxide improves the wettability of the material and provides additional pseudo-capacitance that leads to enhanced energy and power densities (49.93 Wh kg^−1^ and 743.11 W kg^−1^). Our results illustrate that by slightly modifying the structure and composition of carbon-derived materials through a simple and environmentally friendly method, electrode materials exhibiting high-energy-density SCs could be produced. The synergistic effect provided by both hetero-atom-doped carbon and with bimetallic oxides paves way for increasing the energy density of SCs while maintaining their power density. Obviously, this strategy could be adopted to fabricate electrochemically active materials that find applications in various fields including in supercapacitors, sensors and so on.

## Data Availability

Data are contained within the article and Supplementary Materials.

## Supplementary Materials

The following supporting information can be downloaded at: https://www.mdpi.com/article/10.3390/polym16030430/s1, Figure S1. (a) XRD; (b) Raman; (c) BET; and (d) pore size distribution of HC/NiCo at different calcination temperatures. Figure S2. XPS spectra of HC/NiCo@800 showing the (a) survey spectrum; deconvolution spectrum of (b) C1s; (c) N 1s; (d) O 1s; (e) Co 2p and (f) Ni 2p. Figure S3. SEM images of HC/NiCo@800 after stability test. Figure S4. CV curves of (a) HC and (b) HC/NiCo@800 in three-electrode system. The details regarding the instrumentation, electrochemical measurements and structural characterization of the materials are given in the supplementary information. References [10,12,14,16,17,18,29,30,32,33,34,35,36,37,38,39] are cited in the supplementary materials.
