# Supplementary material for: Polybenzoxazine-Based Nitrogen-Containing Porous Carbon and Their Composites with NiCo Bimetallic Oxides for Supercapacitor Applications
Source: Polymers (Basel). 2024 Feb 3;16(3):430. doi: 10.3390/polym16030430 (PMC10857209; doi:10.3390/polym16030430)
Supplement: Supplementary file 1 [file polymers-16-00430-s001.zip › polymers-2823812-supplementary.pdf]

Supplementary Information

Polybenzoxazine-based nitrogen-containing porous carbon and their composites with NiCo for supercapacitor applications

1. Instrumentation

The prepared HC/NiCo materials were characterized by various physicochemical techniques such as field emission scanning electron microscopy (FESEM) with energy-dispersive X-ray spectroscopy (EDS), high-resolution transmittance electron microscopy (HRTEM), X-ray diffraction (XRD), Raman spectroscopy, nitrogen adsorption-desorption isotherms, and X-ray photoelectron spectroscopy (XPS). FESEM with EDS analysis was carried out on a Hitachi S-4800 equipped with EDX at an accelerating voltage of 4 kV. HRTEM images were performed with an FEI-Tecnai TF-20 transmission electron microscope with an operating accelerating voltage of 120 kV. XRD measurements were carried out using a PANalytical X’Pert^3 MRD diffractometer with monochromatized Cu Kα radiation (λ = 1.54 Å) at 40 kV and 30 mA and were recorded in the range from 10 to 90° (2θ). Raman spectrum was recorded on the XploRA Micro-Raman spectrophotometer (Horiba) with the range between 50 and 4000 cm^{-1}. Nitrogen sorption isotherms were measured at −197 °C using a Micromeritics ASAP 2000. Before the experiments, the samples were dried at 120 °C and evacuated for 8 h in flowing argon at the flow rate of 60 standard cubic centimeters per minute at 140 °C. Surface area, pore size, and pore volumes were obtained from isotherms using the conventional Brunauer-Emmet-Teller (BET) and Barrett-Joyner-Halenda (BJH) equations. XPS spectra were achieved using a K-Alpha (Thermo Scientific). CasaXPS software was used for the deconvolution of the high-resolution XPS spectra.

2. Electrochemical measurements

All electrochemical measurements including cyclic voltammetry (CV), galvanostatic charge-discharge (GCD), and electrochemical impedance spectroscopy (EIS) were conducted with a typical three-electrode system and were performed on the CorrTest-CS350 electrochemical workstation in 1 M KOH aqueous solution. A commercial Hg/HgO electrode was employed as a reference electrode, a platinum plate (1 cm^2) was used as a counter electrode and HC/NiCo material loaded nickel form (HC/NiCo) was used as working electrodes. The CV measurements were carried out at a potential window from 0.0 and 0.5 V (*vs*. Hg/HgO) under the different scan rates from 5 to 200 mV s^{-1}). The GCD measurements were performed with a potential window of 0.0−0.5 V (*vs*. Hg/HgO) at the current densities varied from 1 to 10 A g^{-1}. EIS measurements were performed in the frequency range of 0.01 kHz – 100 kHz with an alternating current amplitude of 5 mV. All the electrochemical tests were conducted at room temperature. The capacitances of the electroactive materials were obtained from their GCD curves according to the following equation [1].

$$C_s = \frac{I\Delta t}{m\Delta V} \quad \text{.........} \ [1]$$

whereas, C_s is the specific capacitances (F g^{-1}), I is the current in the charge-discharge process (A), Δt represents to the discharge time (s), ΔV stands for the potential window during the charge-discharge measurement (V) and m donates the mass of the electroactive materials (g).

3. Results and discussion

3.1. XRD

The phase purity and crystallinity of HC/NiCo at different calcination temperatures were characterized by XRD analysis. Figure S1a shows the XRD patterns of HC/NiCo calcined at

different temperatures. As shown in the figure, all the diffraction peaks are assigned to the cubic planes of NiCo [(111), (220), (311), (400), (511) and (620)] at different 2θ values [JCPDS no. 73-1702]. The diffraction peak between 15 – 30° is assigned to the characteristic (002) plane of the hexagonal graphitic structure of carbon. Generally, a broad peak is obtained in the above said region, due to the amorphous nature of carbon. This broad peak is disturbed due to the inclusion of bimetallic elements into the carbon framework, resulting in dis-ordered arrangement and lowered graphitization degree [1-4].

3.2. Raman

The Raman spectra of HC/NiCo at different calcination temperature is shown in Fig. S1b. The two typical fingerprint regions, representing the characteristic D-band and G-band of carbon materials are observed at 1336 and 1575 cm^{-1}, respectively. The R value, i.e., I_D/I_G peak ratio is used to evaluate the degree of graphitization of carbon materials. The calculated peak intensities between D and G bands were found to be 1.12, 0.93, 0.93 and 0.96 for HC/NiCo@600, HC/NiCo@700, HC/NiCo@800 and HC/NiCo@900, respectively. This R value indicates that there are more structural defects in the carbon material due to the inclusion of bimetallic oxides. In addition to this, a broad peak centered at 619 cm^{-1} was found in all the spectrum, due to the formation of metal-oxygen-metal bond [3, 5-6]. It could be observed that the intensity of this peak is more pronounced and broad in HC/NiCo@800 when compared with the other. This shows that 800 ℃ is the most suitable temperature to form the bimetallic oxides in the carbon interstices.

3.3. BET analysis

The porosity of the prepared materials including pore size, pore volume and surface area was investigated using BET analysis. Figure S1c & d depicts the N_2 adsorption/desorption isotherms and pore size distribution (PSD) of the prepared materials. A typical type IV isotherm is observed

(Fig. S1c) between $0.5 < P/P_0 < 0.9$ for all the prepared materials, indicating the presence of mesopores in their structure. Moreover, there is an overlap of adsorption and desorption isotherms, which indicates the co-existence of micro-pores and meso-pores in the structure [3, 7-8]. The PSD result indicates that the majority of pores are between 10 – 50 nm in diameter, for all the prepared materials (Fig. S1d), in which, HC/NiCo@800 contains enormous amount of pores between 3 – 12 nm and 26 nm in diameter. The HC/NiCo material prepared at a calcination temperature of 800 ℃ shows the proper distribution of pores in the micro-porous and meso-porous region. The BET surface area of HC/NiCo@600, HC/NiCo@700, HC/NiCo@800 and HC/NiCo@900 was found to be 224, 235, 286 and 240 m^2g^{-1}, respectively. The results indicate that the calcination temperature of 800 ℃ is suitable to produce unique hierarchical porous structure with appropriate surface area that could facilitate adsorption and desorption of ions at a faster rate with proper mechanical integrity [2, 3].

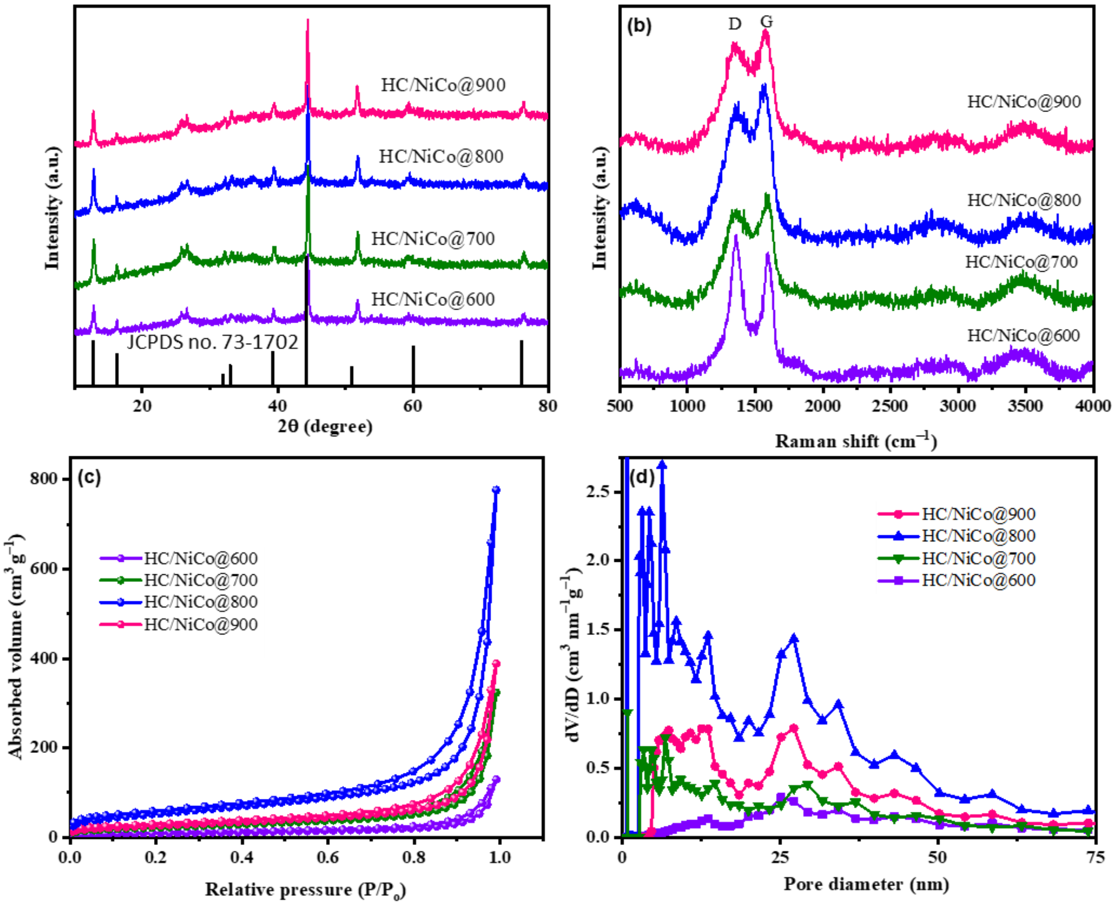

Figure S1. (a) XRD; (b) Raman; (c) BET; and (d) pore size distribution of HC/NiCo at different calcination temperatures.

3.4. XPS analysis

The surface chemical composition and valence state of HC/NiCo@800 were further analyzed by XPS and displayed in Fig. S2. Figure S2a shows the survey spectrum and Figs. S2b–f show the deconvoluted spectrum of HC/NiCo@800. The survey spectrum (Fig. S2a) confirms the presence of C, N, O, Co and Ni elements. The intensity of O 1s is higher than N 1s, indicating that oxygen

atoms could be obtained both from the carbon surface and through the formation of bimetallic oxides [3]. Each spectrum of the respective element(s) is deconvoluted for more detailed analysis. The high resolution C 1s spectrum is deconvoluted into four different peaks (Fig. S2b). The peak at 284.2 eV is due to the C=C carbon of the aromatic ring; the peak at 285.4 eV is due to the C–C single bond; the peak at 287.5 eV is due to the carbonyl carbon (C=O/C–O); and the peak at 290.6 eV is due to the C–N carbon. Similarly, the N 1s spectrum is deconvoluted into three different peaks (Fig. S2c). The nitrogen atom is derived only form the carbon part that exist in different forms showing pyridinic N at 397.8 eV, pyrrolic N at 399.6 eV and quaternary N at 401.3 eV. The presence of different forms of nitrogen species could enhance the capacitance, as pyridinic and pyrrolic N involve in the pseudo-capacitive interactions, and quaternary N will increase the conductivity of the carbon material through effective ion transfer. The O 1s spectrum is deconvoluted into three different peaks (Fig. S2d). The peak at 530.7 eV with high intensity is due to the formation of metal-oxygen bonds. The other peaks with lower intensities at 531.9 and 533.1 eV are due to the formation of surface adsorbed hydroxyl (–OH) and carbonyl (C–O) oxygens. The presence of oxygen atoms can bring about wettability of the electrode materials and thus enhance the overall capacitance [9-13].

The deconvoluted spectrum of Co 2p (Fig. S2e) shows the splitting of two spin states of Co at 781.6 and 797.4 eV that belongs to the spin-orbit splitting values of Co $2p_{3/2}$ and Co $2p_{1/2}$, respectively. Moreover, the satellite peaks were also observed indicating the co-existence of Co^{2+} and Co^{3+}. The Ni 2p deconvoluted spectrum (Fig. S2f) shows two spin-orbit doublets at 854.8 and 873.1 eV, corresponding to Ni $2p_{3/2}$ and Ni $2p_{1/2}$ spin states. Furthermore, the two satellite bands indicates the existence of Ni^{2+} state. The presence of bimetallic components with multiple valence states could facilitate rapid redox reactions and enhance the pseudo-capacitance [4, 7, 14-16]. All

the above mentioned results indicate the successful incorporation of bimetallic oxide into the carbon framework.

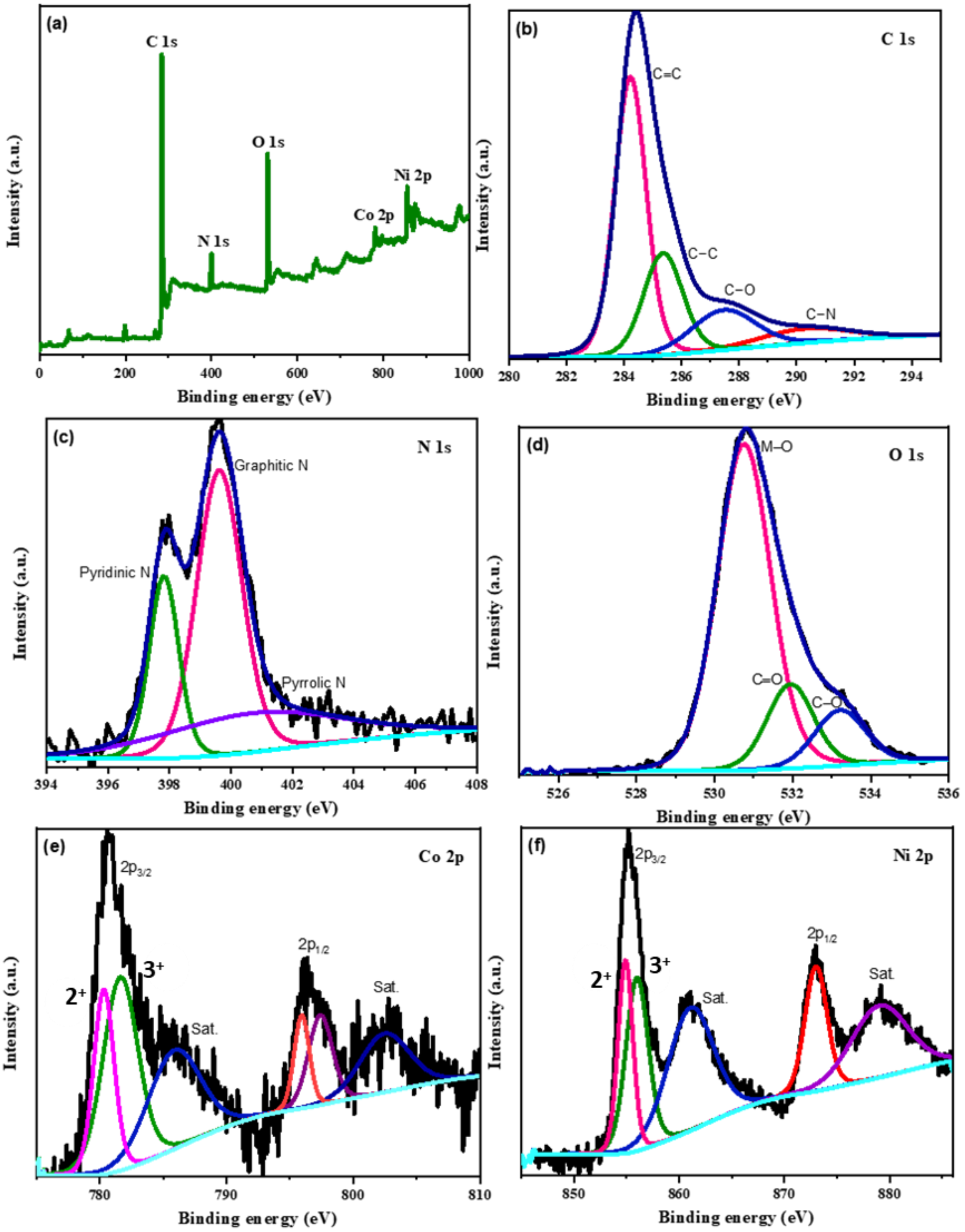

Figure S2. XPS spectra of HC/NiCo@800 showing the (a) survey spectrum; deconvolution spectrum of (b) C1s; (c) N 1s; (d) O 1s; (e) Co 2p and (f) Ni 2p.

3.5. SEM analysis after stability test

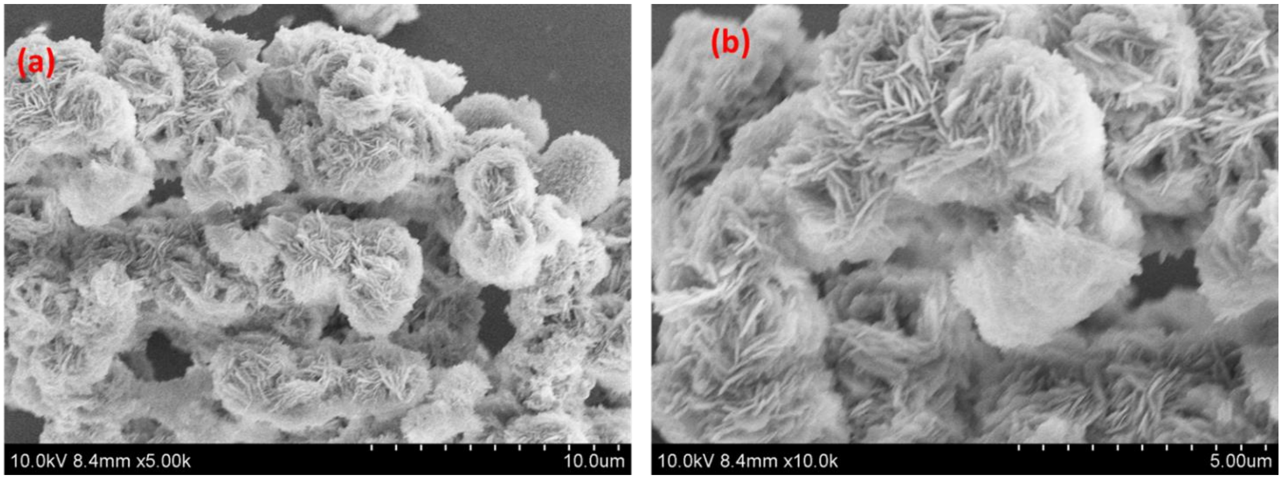

Figure S3. SEM images of HC/NiCo@800 after stability test

3.6. CV measurement of negative and positive electrode

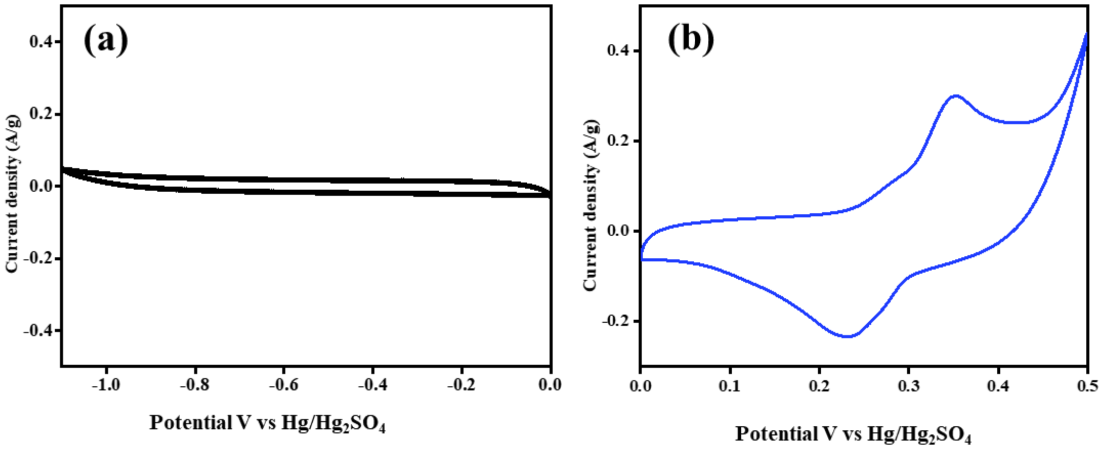

Figure S4. CV curves of (a) HC and (b) HC/NiCo@800 in three-electrode system

References

1. Li, L.; Cheah, Y.; Ko, Y.; Teh, P.; Wee, G.; Wong, C.; Peng, S.; Srinivasan, M. The Facile Synthesis of Hierarchical Porous Flower-like NiCo 2O4 with Superior Lithium Storage Properties. *J. Mater. Chem. A* **2013**, *1* (36), 10935–10941. https://doi.org/10.1039/c3ta11549f.
2. Zhang, J.; Luo, J.; Guo, Z.; Liu, Z.; Duan, C.; Dou, S.; Yuan, Q.; Liu, P.; Ji, K.; Zeng, C.; Xu, J.; Liu, W. Di; Chen, Y.; Hu, W. Ultrafast Manufacturing of Ultrafine Structure to Achieve An Energy Density of Over 120 Wh Kg−1 in Supercapacitors. *Adv. Energy Mater.* **2023**, *13* (1), 1–9. https://doi.org/10.1002/aenm.202203061.
3. Zhao, J.; Chen, J.; Xu, S.; Shao, M.; Zhang, Q.; Wei, F.; Ma, J.; Wei, M.; Evans, D. G.; Duan, X. Hierarchical NiMn Layered Double Hydroxide/Carbon Nanotubes Architecture with Superb Energy Density for Flexible Supercapacitors. *Adv. Funct. Mater.* **2014**, *24* (20), 2938–2946. https://doi.org/10.1002/adfm.201303638.
4. Chen, K.; Zhao, S.; Sun, J.; Zhou, J.; Wang, Y.; Tao, K.; Xiao, X.; Han, L. Enhanced Capacitance Performance by Coupling 2D Conductive Metal-Organic Frameworks and Conducting Polymers for Hybrid Supercapacitors. *ACS Appl. Energy Mater.* **2021**, *4* (9), 9534–9541. https://doi.org/10.1021/acsaem.1c01694.
5. Beidaghi, M.; Wang, C. Micro-Supercapacitors Based on Interdigital Electrodes of Reduced Graphene Oxide and Carbon Nanotube Composites with Ultrahigh Power Handling Performance. *Adv. Funct. Mater.* **2012**, *22* (21), 4501–4510. https://doi.org/10.1002/adfm.201201292.
6. Fan, Z. J.; Yan, J.; Wei, T.; Ning, G. Q.; Zhi, L. J.; Liu, J. C.; Cao, D. X.; Wang, G. L.; Wei, F. Nanographene-Constructed Carbon Nanofibers Grown on Graphene Sheets by Chemical

Vapor Deposition: High-Performance Anode Materials for Lithium Ion Batteries. *ACS Nano* **2011**, *5* (4), 2787–2794. https://doi.org/10.1021/nn200195k.

7. Ren, C.; Jia, X.; Zhang, W.; Hou, D.; Xia, Z.; Huang, D.; Hu, J.; Chen, S.; Gao, S. Hierarchical Porous Integrated Co1−xS/CoFe2O4@rGO Nanoflowers Fabricated via Temperature-Controlled In Situ Calcining Sulfurization of Multivariate CoFe-MOF-74@rGO for High-Performance Supercapacitor. *Adv. Funct. Mater.* **2020**, *30* (45). https://doi.org/10.1002/adfm.202004519.
8. Jayakumar, A.; Antony, R. P.; Wang, R.; Lee, J. M. MOF-Derived Hollow Cage $Ni_xCo_{3-x}O_4$ and Their Synergy with Graphene for Outstanding Supercapacitors. *Small* **2017**, *13* (11), 1603102. https://doi.org/10.1002/smll.201603102.
9. Qu, C.; Zhang, L.; Meng, W.; Liang, Z.; Zhu, B.; Dang, D.; Dai, S.; Zhao, B.; Tabassum, H.; Gao, S.; Zhang, H.; Guo, W.; Zhao, R.; Huang, X.; Liu, M.; Zou, R. MOF-Derived α-NiS Nanorods on Graphene as an Electrode for High-Energy-Density Supercapacitors. *J. Mater. Chem. A* **2018**, *6* (9), 4003–4012. https://doi.org/10.1039/c7ta11100b.
10. Liu, R.; Zhang, A.; Tang, J.; Tian, J.; Huang, W.; Cai, J.; Barrow, C.; Yang, W.; Liu, J. Fabrication of Cobaltosic Oxide Nanoparticle-Doped 3 D MXene/Graphene Hybrid Porous Aerogels for All-Solid-State Supercapacitors. *Chem. - A Eur. J.* **2019**, *25* (21), 5547–5554. https://doi.org/10.1002/chem.201806342.
11. Le, T. A.; Tran, N. Q.; Hong, Y.; Lee, H. Intertwined Titanium Carbide MXene within a 3 D Tangled Polypyrrole Nanowires Matrix for Enhanced Supercapacitor Performances. *Chem. - A Eur. J.* **2019**, *25* (4), 1037–1043. https://doi.org/10.1002/chem.201804291.
12. Guan, C.; Liu, X.; Ren, W.; Li, X.; Cheng, C.; Wang, J. Rational Design of Metal-Organic Framework Derived Hollow NiCo2O4 Arrays for Flexible Supercapacitor and

Electrocatalysis. *Adv. Energy Mater.* **2017**, *7* (12), 1–8. https://doi.org/10.1002/aenm.201602391.

13. Deepalakshmi, T.; Nguyen, T. T.; Kim, N. H.; Chong, K. T.; Lee, J. H. Rational Design of Ultrathin 2D Tin Nickel Selenide Nanosheets for High-Performance Flexible Supercapacitors. *J. Mater. Chem. A* **2019**, *7* (42), 24462–24476. https://doi.org/10.1039/c9ta08677c.
14. Wei, Y. J.; Yan, L. Y.; Wang, C. Z.; Xu, X. G.; Wu, F.; Chen, G. Effects of Ni Doping on [MnO_6] Octahedron in $LiMn_2O_4$. *J. Phys. Chem. B* **2004**, *108* (48), 18547–18551. https://doi.org/10.1021/jp0479522.
15. Liang, J.; Renzhi, M.; Iyi, N.; Ebina, Y.; Takada, K.; Sasaki, T. Topochemical Synthesis, Anion Exchange, and Exfoliation of Co-Ni Layered Double Hydroxides: A Route to Positively Charged Co-Ni Hydroxide Nanosheets with Tunable Composition. *Chem. Mater.* **2010**, *22* (2), 371–378. https://doi.org/10.1021/cm902787u.
16. Ma, R.; Liang, J.; Takada, K.; Sasaki, T. Topochemical Synthesis of Co-Fe Layered Double Hydroxides at Varied Fe/Co Ratios: Unique Intercalation of Triiodide and Its Profound Effect. *J. Am. Chem. Soc.* **2011**, *133* (3), 613–620. https://doi.org/10.1021/ja1087216.
